# Multicenter External Quality Assessment Program for PCR Detection of *Mycobacterium ulcerans* in Clinical and Environmental Specimens

**DOI:** 10.1371/journal.pone.0089407

**Published:** 2014-02-21

**Authors:** Miriam Eddyani, Caroline Lavender, Willem Bram de Rijk, Pieter Bomans, Janet Fyfe, Bouke de Jong, Françoise Portaels

**Affiliations:** 1 Department of Biomedical Sciences, Institute of Tropical Medicine, Antwerp, Belgium; 2 Victorian Infectious Diseases Reference Laboratory, Melbourne, Victoria, Australia; University of Houston, United States of America

## Abstract

**Background:**

*Mycobacterium ulcerans* is the causative agent of Buruli ulcer (BU), a necrotizing disease of the skin, soft tissue and bone. PCR is increasingly used in the diagnosis of BU and in research on the mode of transmission and environmental reservoir of *M. ulcerans*.

**Methodology/Principal Findings:**

The aim of this study was to evaluate the performance of laboratories in detecting *M. ulcerans* using molecular tests in clinical and environmental samples by implementing sequential multicenter external quality assessment (EQA) programs. The second round of the clinical EQA program revealed somewhat improved performance.

**Conclusions/Significance:**

Ongoing EQA programs remain essential and continued participation in future EQA programs by laboratories involved in the molecular testing of clinical and environmental samples for *M. ulcerans* for diagnostic and research purposes is strongly encouraged. Broad participation in such EQA programs also benefits the harmonization of quality in the BU research community and enhances the credibility of advances made in solving the transmission enigma of *M. ulcerans*.

## Introduction

The implementation of PCR-based methods for the detection of *Mycobacterium ulcerans*, the causative organism of Buruli ulcer (BU), in clinical and environmental samples 15 years ago [1]–[3] has drastically improved our knowledge of BU. BU is an indolent necrotizing disease of the skin, subcutaneous tissue, and bone [4] occurring mainly in certain riverine rural areas of West and Central Africa and in coastal southeastern Australia with about 20,000 cases reported in the last decade [4]. BU is presently the third most common mycobacterial disease of humans, after tuberculosis and leprosy, and the least well understood of the three [5].

In most BU endemic settings the working conditions are difficult and the diagnosis of BU is often made on clinical and epidemiological grounds. However, the disease presents with a diverse range of clinical symptoms and, due to possible confusion with other tropical skin diseases, the added value of microbiological confirmation is becoming more appreciated. Among the available laboratory tests (direct smear examination for acid fast bacilli, culture, PCR and histopathology), PCR targeting the insertion element IS*2404* (present in over 200 copies in the *M. ulcerans* genome) is by far the most sensitive and specific, and much faster than culture, which takes an average of 10 weeks and only has 45% sensitivity despite many efforts to improve decontamination methods, culture media and incubation conditions [6]–[10]. Since isolating *M. ulcerans* from environmental sources has until now, despite numerous attempts, only been successful once [11], most current knowledge on the environmental reservoir and mode of transmission of *M. ulcerans* is based on studies that have used PCR to amplify IS*2404* and other targets (less frequent in the *M. ulcerans* genome) such as the insertion element IS*2606*, the ketoreductase B domain (KR) and the enoyl reductase domain (ER) suggesting the presence of *M. ulcerans* DNA in a number of biotic and abiotic elements of the environment [12]–[14]. The actual environmental reservoir(s) and mode(s) of transmission, however, remain a mystery.

Clinical and environmental samples can contain low concentrations of *M. ulcerans* DNA, PCR inhibitors and DNA from other sources that may generate non-specific PCR products, which present a number of difficulties when applying molecular methods to diagnosis and research. Moreover, previous external quality control studies for PCR detection of *Mycobacterium tuberculosis* and hepatitis B virus have shown that PCR may be unreliable because of false-positive results caused by contamination or because of false-negative results caused by a lack of sensitivity, inhibition, or other reasons [15]–[17]. The virtually unique reliance on PCR for diagnostic and research purposes in the field of BU requires the continued and convincing demonstration of its accuracy, reliability and reproducibility. This requirement compels laboratories to establish and implement effective and comprehensive quality assurance schemes for their PCR tests. Quality assurance involves intensive internal quality control as well as a system of external quality control. In light of this, in 2008 the Technical Advisory Group of the WHO Global Buruli Ulcer Initiative recommended the establishment of an external quality assessment program (EQAP) for the molecular detection of *M. ulcerans* in clinical and environmental samples.

EQAPs are performed to assist laboratories maintain high standards. They enable participants to check that samples are processed correctly, that results are appropriately recorded, and that assays are robust and are being performed in an accurate and reproducible manner. External quality assessment can consist of audit visits, proficiency testing with an adequate number of coded specimens and periodic rechecking of specimens. This report summarizes the results of two rounds of proficiency testing for the molecular detection of *M. ulcerans* in clinical and environmental samples coordinated by the WHO Collaborating Centers for *M. ulcerans*, respectively, the Institute of Tropical Medicine (ITM) in Antwerp, Belgium, and the Victorian Infectious Diseases Reference Laboratory (VIDRL) in Melbourne, Australia. The objectives of these programs were to assess the performance of the participating laboratories in detecting *M. ulcerans* DNA and to compare their performance between rounds.

## Materials and Methods

Proficiency testing by both WHO Collaborating Centers involved the distribution to national laboratories of panels of coded specimens with known status, with the coding sequence differing from panel to panel. All samples were shipped at ambient temperature to the participants (Table 1). In addition, a questionnaire on the methodologies used and laboratory characteristics was included in each shipment along with instructions and a results sheet.

**Table 1:** Participating laboratories in the two rounds of clinical and environmental EQAP.

Laboratory	Country	Clinical EQAP		Environmental EQAP	
		round 1	round 2	round 1	round 2
Monash University	Australia	no	no	yes	no
Queensland Mycobacterium Reference Laboratory	Australia	no	yes	no	no
Victorian Infectious Diseases Reference Laboratory	Australia	yes	yes	yes	yes
Institute of Tropical Medicine	Belgium	yes	yes	yes	yes
Laboratoire de Référence des Mycobactéries	Benin	yes	yes	no	no
Centre Pasteur Yaoundé	Cameroon	yes	yes	yes	yes
Institut Pasteur	Côte d’Ivoire	yes	yes	no	yes
Institut de Recherche Biomédicale	DRC	no	yes	no	no
Université d’Angers	France	yes	no	yes	yes
Institut Pasteur Guyane-Cayenne	French Guyana	no	yes	yes	no
Ludwig-Maximilians University Hospital	Germany	yes	yes	no	no
Komfo Anokye Teaching Hospital	Ghana	yes	no	no	no
Kumasi Centre for Collaborative Research	Ghana	no	yes	no	no
Noguchi Memorial Institute for Medical Research 1	Ghana	no	yes	no	yes
Noguchi Memorial Institute for Medical Research 2	Ghana	no	yes	no	no
University of Eastern Piedmont	Italy	yes	yes	no	no
National Institute of Infectious Diseases	Japan	no	yes	yes	no
Institut Pasteur	Central African Republic	yes	yes	no	no
Swiss Tropical and Public Health Institute	Switzerland	no	yes	no	yes
Institut National d’Hygiene	Togo	no	yes	no	no
University of Tennessee	USA	yes	yes	yes	yes

Participating laboratories were asked to process the EQA panel using the DNA extraction and PCR methods they routinely used for molecular detection of *M. ulcerans*. To ensure confidentiality, all participating laboratories were assigned a code. The test results by the laboratories were compared with the coded results in a blinded way and specific performance indicators (concordance, sensitivity, specificity and reproducibility) calculated. The participating laboratories received a report summarizing the results of the EQAP that allowed them to compare their performance to that of other laboratories. Individual discussions between the WHO Collaborating Centers and some participants to reflects on possible causes and solutions of weak performances took place by email and at the WHO BU meeting in Geneva, Switzerland in March 2010.

### Ethics Statement

The existing collection of anonymized surplus diagnostic samples hosted by the ITM in its role of WHO Collaborating Centre for the Diagnosis and Surveillance of *Mycobacterium ulcerans* Infection was used to prepare the distributed EQA panels. For this kind of activity no approval of an ethics committee is needed according to the Belgian law of 7 May 2004 concerning experiments on humans (Chapter II, Art. 2, 23′ and Chapter II, Art. 3) [18].

### Clinical EQAP

During the first round, organized in 2009, the EQA panel for the detection of *M. ulcerans* DNA consisted of 34 suspensions of clinical specimens in PBS and 70% ethanol (50∶50) to kill bacteria. During the second round, organized in 2011, 33 such suspensions were included. Suspensions were selected in such a way that they would allow an assessment of sensitivity, specificity, and inter-laboratory reproducibility (Table 2). The positive suspensions represented strong as well as weak positives (quantified by direct smear examination). Among the *M. ulcerans* negative samples, some suspensions of clinical specimens positive for *M. tuberculosis* and *M. marinum* were included. All suspensions were sent in duplicate to assess intra-laboratory reproducibility. In order to distinguish whether weak performance during the first round was due to problems during DNA extraction versus amplification, serial dilutions of extracted genomic *M. ulcerans* DNA suspended in 1xTE were distributed during the second round, in addition to the suspensions. Ten-fold dilutions of 29×10^−4^ ng/µl to 29×10^−9^ ng/µl genomic DNA were included in duplicate. Testing both specimen suspensions and DNA extracts allowed laboratories to evaluate the performance of their methods for DNA extraction and for PCR, separately.

**Table 2:** Panel composition of the two rounds of clinical EQAP and concordant qualitative results per sample (inter-laboratory reproducibility).

round 1 clinical EQAP				round 2 clinical EQAP			
Sample ID	Sample status (IS2404-Ct value)^a^	Direct smear examination	N° of concordant results/Total n° of respondent laboratories (%)^b^	Sample ID	Sample status (IS2404-Ct value)^a^	Direct smear examination	N° of concordant results/Total n° of respondent laboratories (%)^b^
4	Positive (34.47)	Negative	9/11 (82)	1	Negative	Negative	14/15^b^ (93)
44	Positive (34.15)	Negative	7/11 (64)	2	Negative	Negative	16/16 (100)
117	Negative	Negative	10/11 (91)	3	*M. tuberculosis*	Negative	16/16 (100)
140	Negative	Negative	10/11 (91)	4	Positive (29.49)	4+	13/16 (81)
172	Negative	Negative	8/11 (73)	5	Negative	Negative	16/16 (100)
231	Negative	Negative	8/10 (80)	6	Positive (37.33)	Negative	12/16 (75)
269	*M. tuberculosis*	1+	10/11 (91)	7	Positive (22.14)	4+	14/16 (88)
299	*M. tuberculosis*	1+	10/11 (91)	8	Positive (38,69)	Negative	9/16 (56)
318	*M. tuberculosis*	Negative	8/11 (73)	9	Positive (30.27)	1+	16/16 (100)
387	*M. tuberculosis*	Negative	10/11 (91)	10	Negative	Negative	16/16 (100)
433	Positive (28.00)	2+	9/11 (82)	11	*M. marinum*	Negative	16/16 (100)
457	Positive (28.13)	2+	10/11 (91)	12	Negative	Negative	14/16 (88)
502	Negative	Negative	8/11 (73)	13	Negative	Negative	15/16 (94)
545	Negative	Negative	9/11 (82)	14	Negative	Negative	15/16 (94)
631	Negative	Negative	7/11 (64)	15	Positive (19.90)	4+	16/16 (100)
654	Negative	Negative	7/11 (64)	16	Negative	Negative	16/16 (100)
681	*M. tuberculosis*	Negative	10/11 (91)	17	Positive (38.43)	Negative	9/16 (56)
741	*M. tuberculosis*	Negative	10/11 (91)	18	Negative	Negative	14/16 (88)
787	Positive (22.35)	4+	10/11 (91)	19	Negative	Negative	14/16 (88)
864	Positive (22.88)	4+	10/11 (91)	20	*M. tuberculosis*	Negative	15/16 (94)
908	Positive (37.78)	Negative	4/9 (44)	21	Positive (29.41)	4+	13/16 (81)
962	Positive (36.80)	Negative	5/10 (50)	22	Negative	Negative	15/16 (94)
1017	Positive (22.10)	4+	11/11 (100)	23	Positive (37.30)	Negative	11/16 (69)
1117	Positive (22.10)	4+	11/11 (100)	25	Positive (38.92)	Negative	5/16 (31)
1185	Negative	Negative	10/11 (91)	26	Positive (29.34)	1+	14/16 (88)
1212	Negative	Negative	9/11 (82)	27	Negative	Negative	16/16 (100)
1271	Positive (36.44)	Negative	6/10 (60)	28	*M. marinum*	Negative	15/16 (94)
1283	Positive (36.70)	negative	7/11 (64)	29	Negative	Negative	15/16 (94)
1366	Negative	1+	9/10 (90)	30	Negative	Negative	16/16 (100)
1391	Negative	1+	8/10 (80)	31	Negative	Negative	16/16 (100)
1475	Negative	Negative	11/11 (100)	32	Positive (19.92)	4+	13/16 (81)
1505	Negative	Negative	10/11 (91)	33	Negative	Negative	15/16 (94)
1578	Positive (32.56)	1+	10/11 (91)	34	Positive (37.45)	Negative	7/16 (44)
1649	Positive (32.36)	1+	10/11 (91)				

a No sample was inhibited. ^b^ The total number of respondent laboratories varies because inconclusive results were removed from the analysis as well as a missing suspension in one laboratory in round 1.

### Environmental EQAP

During the first (pilot) round, organized in 2008, participants were sent eight heat-sterilized environmental samples each comprising a mixture of soil, leaf litter, detritus and animal faeces collected from BU endemic and non-endemic areas in Victoria, Australia. The positive samples were true positives containing varying concentrations of *M. ulcerans* DNA as determined by IS*2404* real-time PCR [14] (Table 3). The rationale for using only one sample type during the pilot round was to minimize the number of variables so that the only difference between samples was the presence or absence of *M. ulcerans* DNA. During the second round, organized in 2010, participants received 10 heat-sterilized environmental samples. The samples were selected to represent the types of samples commonly tested by BU researchers (water, soil, aquatic plants and animal faeces) and to include the types of samples that often contain PCR inhibitors (Table 3). The positive samples represented stronger as well as weaker positives as determined by IS*2404* real-time PCR [14] (Table 3). The positive faecal samples were true positive samples collected from a BU endemic area, whereas the other positive samples were spiked with suspensions of *M. ulcerans*, as in Australia these types of samples are less frequently positive and contain lower concentrations of *M. ulcerans* DNA [19], rendering the collection of true positive samples difficult. To assess the impact of a delay in the shipment and/or processing of samples, two panels were retained at VIDRL (one stored at 4°C and the other at room temperature) and tested after all participants had submitted their results.

**Table 3:** Panel composition of the two rounds of environmental EQAP and concordant results per sample (inter-laboratory reproducibility).

round 1 environmental EQAP					round 2 environmental EQAP				
Sample ID	Sampletype^a^	Sample status (IS2404-Ctvalue before, after)^b^	N° of concordant results/Total n° of respondent laboratories (%)^c^	Sample ID		Sample type	Sample status (IS2404-Ctvalue before, after)^b^	N° of concordant results/Total n°of respondent laboratories (%)	
1	Mixture	Positive (26.53, 27.74)	7/7 (100)	1		Soil	Negative	8/8 (100)	
2	Mixture	Negative	6/7 (86)	2		Soil	Positive (22.19, 23.11)	6/8 (75)	
3	Mixture	Negative	6/7 (86)	3		Water	Negative	7/8 (88)	
4	Mixture	Positive (25.73, 27.22)	7/7 (100)	4		Algae	Negative	8/8 (100)	
5	Mixture	Positive (23.64, 25.13)	7/7 (100)	5		Faeces	Negative	7/8 (88)	
6	Mixture	Negative	6/7 (86)	6		Algae	Positive (28.89, 35.25)	7/8 (88)	
7	Mixture	Negative	6/7 (100)	7		Faeces	Positive (33.77, 34.09)	5/8 (63)	
8	Mixture	Negative	6/6 (100)	8		Water	Positive (36.19, 37.54)	8/8 (100)	
				9		Faeces	Negative	7/8 (88)	
				10		Faeces	Positive (30.48, 30.49)	6/8 (75)	

a All samples consisted of a mixture of soil, leaf litter, detritus, animal faeces.

b Mean Ct value of duplicate IS2404 real-time PCR before samples were shipped to participants and after all participants had returned results.

c One laboratory was missing a sample in round 1.

### Statistical Analysis

Inter-laboratory reproducibility was calculated per sample as the number of qualitative results concordant with the organizing centers obtained by the laboratories divided by the total number of participating laboratories.

The concordance of a laboratory was calculated as the ratio of concordant qualitative results obtained by the laboratory over the total number of samples.

Results were considered false negative or false positive when they differed from the qualitative results obtained by the organizing laboratory using real-time PCR.

Intra-laboratory reproducibility was assessed by shipping all suspensions in duplicate and calculated as the ratio of pairs concordant with the organizing centers over the total number of pairs.

GraphPad Prism v. 5 was used for linear regression analysis of reproducibility and concordance vs. workload, and false negative rate vs. the PCR detection limit.

## Results

### Clinical EQAP

A total of 11 laboratories from 11 countries participated in the first round of the clinical EQAP while 18 laboratories from 15 countries took part in the second round (Table 1).

#### Results by sample (inter-laboratory reproducibility)

During the first round, the proportion of qualitative results concordant with ITM varied between 44% and 100%, median 90%, by sample (Table 2). Only 3 samples were identified correctly by all laboratories. In the second round, this proportion ranged between 31% and 100%, median 94%, with eleven samples identified correctly by all laboratories. Six suspensions (actually the duplicates of three) were reported correctly by less than 60% of the laboratories indicating that they were positive at the detection limit of the PCR assays used. These samples were indeed negative by direct smear examination and had reported IS*2404*-Ct values between 36 and 37, as compared with Ct values between 20 and 30 for smear positive samples.

#### Results by laboratory (concordance, false positive and false negative results, intra-laboratory reproducibility)

The proportion of concordant qualitative results varied between 58% and 100%, median 82%, by laboratory in the first round and between 64% and 100%, median 88%, in the second round (Table 4). In both rounds only two laboratories reported all results correctly.

**Table 4:** Qualitative results per laboratory of the two rounds of clinical EQAP.

round 1 clinical EQAP											
Laboratory code	N° of concordant results/Total n° of suspensions analysed (%)^a^	N° of false positives (%)	N° of false negatives? (%)	Intra-laboratory reproducibility^b^	Delay in analysis (days)^c^	Laboratory type	N° clinical specimens tested in 2008	Extraction method	PCR assay	PCR target	
1	33/33 (100)	0 (0)	0 (0)	100	26	Reference	100–499	commercial	real-time	IS*2404*	
3	25/34 (74)	9 (45)	0 (0)	59	7	Reference	>500	in-house	nested	IS*2404*	
4	30/32 (94)	0 (0)	2 (14)	100	137	Reference	100–499	commercial	single	IS*2404*	
5	22/32 (69)	6 (32)	4 (31)	73	140	Hospital	100–499	in-house	single	IS*2404*	
8	26/34 (76)	2 (10)	6 (43)	65	83	Reference	1–29	in-house	single	IS*2404*	
10	19/33 (58)	6 (32)	8 (57)	88	160	Academic	1–29	in-house	single and real-time	IS*2404* and ER	
13	28/34 (82)	4 (20)	2 (14)	76	74	Academic	30–99	commercial	nested	IS*2404* and IS*2606*	
17	34/34 (100)	0 (0)	0 (0)	100	42	Reference	>500	commercial	single	IS*2404*	
18	29/33 (88)	3 (16)	0 (0)	100	176	Hospital	>500	in-house	real-time	IS*2404* and KR	
19	33/34 (97)	1 (5)	0 (0)	94	5	Reference	>500	in-house	nested	IS*2404*	
20	23/34 (68)	0 (0)	11 (79)	82	12	Academic	100–499	commercial	single	IS*2404*	
**round 2 clinical EQAP**											
**Laboratory code**	**N° of concordant results/Total n° of suspensions analyzed (%)** a	**N° of false positives (%)**	**N° of false negatives (%)**	**Intra-laboratory reproducibility^b^**	**Delay in analysis (days)^c^**	**Laboratory type**	**N° clinical specimens tested in 2010**	**Extraction method**	**PCR assay**	**PCR target**	**Lowest DNA dilution detected (x29 ng/µl)**
1	32/32 (100)	0 (0)	0 (0)	100	27	Reference	100–499	commercial	real-time	IS*2404*	10^−8^
2	26/33 (79)	4 (20)	3 (23)	81	16	Reference	100–499	commercial	real-time	IS*2404*	10^−7^
3	30/33 (91)	2 (10)	1 (8)	81	40	Private	1–29	commercial	real-time	IS*2404*	10^−7^
5	23/33 (70)	0 (0)	10 (77)	94	40	Reference	1–29	in-house	single	IS*2404*	10^−5^
6	32/33 (97)	0 (0)	1 (8)	94	89	Academic	>500	commercial	single	IS*2404*	10^−7^
7	33/33 (100)	0 (0)	0 (0)	100	22	Academic	100–499	commercial	real-time	IS*2404*	10^−8^
8	29/33 (88)	0 (0)	4 (31)	88	36	Academic	0	in-house	real-time	IS*2404* and ER	10^−6^
9	NT	NT	NT	NT	20	Academic	1–29	commercial	single	IS*2404*	10^−6^
10	28/33 (85)	0 (0)	5 (38)	94	49	Academic	100–499	commercial	nested	IS*2404* and IS*2606*	Nothing detected
13	28/33 (85)	0 (0)	5 (38)	94	35	Academic	100–499	commercial	single	IS*2404*	10^−7^
14	29/33 (88)	3 (15)	1 (8)	75	33	Reference	1–29	commercial	nested	IS*2404*	10^−7^
15	21/33 (64)	0 (0)	12 (92)	94	48	Reference	0	commercial	single	IS*2404*	10^−5^
19	32/33 (97)	1 (5)	0 (0)	94	60	Reference	1–29	commercial	real-time	IS*2404*	10^−8^
22	NT	NT	NT	NT	40	Reference	100–499	commercial	single	IS2*404*	10^−7^
23	26/33 (79)	2 (10)	5 (38)	81	40	Reference	100–499	commercial	nested	IS*2404*	10^−7^
25	29/33 (88)	0 (0)	4 (31)	100	17	Academic	100–499	in-house	nested	IS*2404*	10^−7^
26	27/33 (82)	0 (0)	6 (46)	88	41	Reference	100–499	commercial	real-time	IS*2404*	10^−6^
28	29/33 (88)	2 (10)	2 (15)	75	160	Reference	100–499	in-house	nested	IS*2404*	10^−8^

a The total n° of suspensions analyzed varies per laboratory because some laboratories reported inconclusive results. ^b^ The number of concordant pairs divided by the total number of pairs. ^c^ Delay between the date of shipment of the panel and the reported date of analysis. NT: not tested; ER: enoyl reductase domain; KR: ketoreductase B domain.

In the first round, seven (64%) of the participating laboratories reported false positive results while six (55%) laboratories reported false negative results. Five laboratories tested for individual inhibition and one of them erroneously observed inhibition in two samples. In the second round, six (38%) participating laboratories reported false positive results while 13 (81%) reported false negative results. Seven laboratories tested for inhibition in individual samples and correctly observed none.

Intra-laboratory reproducibility varied between 59 and 100%, median 88%, and was less than 90% in six laboratories (55%) during the first round (Table 4). During the second round reproducibility within laboratories varied between 75% and 100%, median 94%, and was less than 90% in seven laboratories (44%).

#### Laboratory type, workload and methods used

In 2009, reference laboratories (n = 6) reported between 74% and 100%, median 95%, concordant qualitative results and achieved intra-laboratory reproducibilities between 59% and 100%, median 97%. Academic and hospital laboratories (n = 5) reported between 58% and 87%, median 69%, concordant results and intra-laboratory reproducibilities between 73% and 100%, median 82%.

In 2011, reference laboratories (n = 9) reported between 64% and 100%, median 82%, concordant qualitative results and achieved an intra-laboratory reproducibility between 75% and 100%, median 88%. Academic and private laboratories (n = 7) reported between 85% and 100%, median 88%, concordant results and an intra-laboratory reproducibility between 81% and 100%, median 94%.

**Figure 1:**
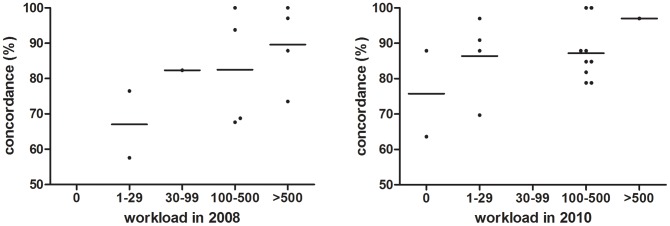
Concordance expressed as proportion of concordant results of participating laboratories vs. workload expressed as number of clinical samples processed in 2008 (A) and in 2010 (B). A linear regression analysis identified a non-significant trend towards better performance by laboratories handling high sample volumes (A: p = 0,0830; B: p = 0,1618).

**Figure 2:**
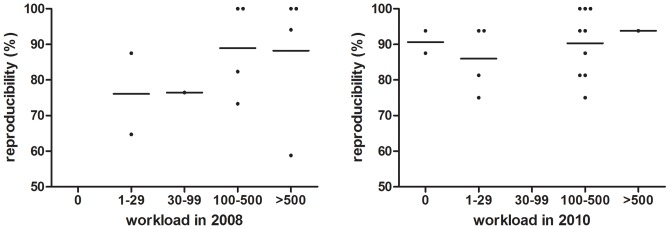
Intra-laboratory reproducibility expressed as the ratio of correctly analysed clinical sample pairs over the total number of pairs versus workload expressed as number of clinical samples processed in 2008 (A) and in 2010 (B). A linear regression analysis indicated that the slope of both graphs does not significantly deviate from zero (A: p = 0,3183; B: p = 0,5623).

When stratifying for workload we found a non-significant correlation between workload and concordance (Fig. 1) and no association between workload and reproducibility (Fig. 2).

Participants used a range of DNA extraction protocols, PCR methods and amplification targets, including both in-house methods (Phenol-chloroform and modified Boom [20]) and kits (e.g. Roche respiratory specimen preparation kit, Qiagen QiAmp DNA mini kit, MOBID ultraclean soil kit and MoBio spin columns, Qiagen Puregene core kit, Qiagen DNeasy blood and tissue kit, Roche high pure PCR template preparation kit, Promega Maxwell 16 Instrument) for DNA extraction and gel-based [21]–[23] and real-time PCR [14], [24] for sequence detection. In the first round, six laboratories reported the use of an in-house DNA extraction method (58%–97%, median 75% concordant results) while five reported to use a commercial method (68%–100%, median 94% concordant results). Three laboratories used real-time PCR (58%–100%, median 88% concordant results). Among the eight laboratories using gel-based conventional PCR, three used a nested format (74%–97%, median 82% concordant results) while five used a single run format (68%–100%, median 77% concordant results). All laboratories amplified the IS*2404* sequence. One laboratory amplified additionally also the gene encoding for ketoreductase (KR), one laboratory the gene encoding for enoyl reductase (ER), while a third laboratory amplified also the IS*2606* sequence.

In the second round, four laboratories reported the use of an in-house DNA extraction method (70%–88%, median 88% concordant results) while 12 reported to use a commercial method (64%–100%, median 86% concordant results). Seven laboratories used real-time PCR (79%–100%, median 91% concordant results). Among the nine laboratories using gel-based conventional PCR, five used a nested format (79%–88%, median 88% concordant results) while four used a single run format (64%–97%, median 77% concordant results). All laboratories amplified the IS*2404* sequence. One laboratory amplified additionally the gene encoding for enoyl reductase (ER), while a second laboratory also amplified the IS*2606* sequence.

#### DNA extracts

**Figure 3:**
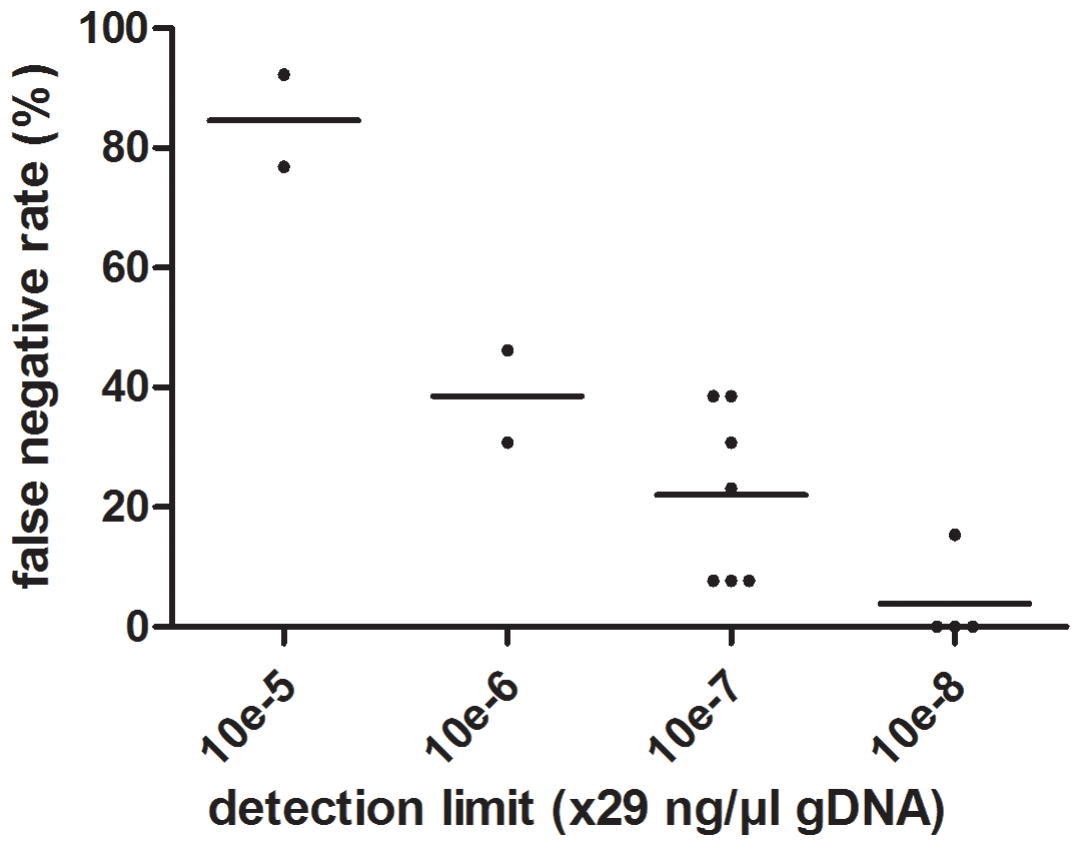
A significant correlation exists between the rate of false negative clinical samples versus the detection limit of the PCR-assay (p<0.0001, R^2^ = 0,7959).

All laboratories but one were able to detect 10^−5^*29 ng/µl or less DNA. No laboratory detected the lowest dilution of 10^−9^*29 ng/µl. One laboratory detected *M. ulcerans* DNA in none of the DNA dilutions. Laboratories able to detect lower concentrations of DNA also had less false negative results among the specimen suspensions (Fig. 3) and this association was significant (p<0.0001).

### Environmental EQAP

Seven laboratories from six countries participated in the first round of the environmental EQAP (Table 1). Eight laboratories from eight countries took part in the second round (Table 1). Five laboratories participated in both rounds.

#### Results by sample (inter-laboratory reproducibility)

There was a high level of inter-laboratory reproducibility in both rounds (Table 3). In the first round, the proportion of results concordant with VIDRL varied between 86% and 100% by sample, with four samples correctly identified by all laboratories. In the second round, this proportion ranged from 63% to 100%, with three samples correctly identified by all participants.

#### Results by laboratory (concordance, false positive and false negative results)

The proportion of concordant qualitative results varied between 43% and 100% by laboratory in the first round and between 50% and 100% in the second round (Table 5). The median overall performance was 100% concordant in the first round and 95% in the second round. Six laboratories reported 100% concordant results in the first round while four reported 100% concordant results in the second round. Of the five laboratories that participated in both rounds, two reported all results correctly in both rounds, two scored worse in the second round and one scored better.

**Table 5:** Qualitative results per laboratory of the two rounds of environmental EQAP.

Round 1 environmental EQAP												
Laboratory code		N° of concordant results/Total n° of suspensions analysed (%)^a^		N° of false positives (%)		N° of false negatives (%)		Extraction method		PCR assay		PCR target
1		8/8 (100)		0 (0)		0 (0)		Commercial		Real-time		IS*2404*, IS*2606* and KR
2		8/8 (100)		0 (0)		0 (0)		Commercial		Real-time		IS*2404* and KR
3		3/7 (43)		4 (57)		0 (0)		Commercial		Single and nested		IS*2404* and IS*2606*
4		8/8 (100)		0 (0)		0 (0)		Commercial		Single		IS*2404*
5		0/0^b^		N/A		N/A		Not stated		Single and real-time		ER
6		8/8 (100)		0 (0)		0 (0)		Commercial		Real-time		IS*2404*
7		8/8 (100)		0 (0)		0 (0)		Commercial		Real-time		IS*2404* and KR
8		8/8 (100)		0 (0)		0 (0)		In-house		Real-time		IS*2404*, IS*2606* and KR
**Round 2 environmental EQAP**												
**Laboratory code**	**N° of concordant results/Total n° of suspensions analysed (%)** a		**N° of false** **positives (%)**		**N° of false** **negatives (%)**		**N° environmental samples tested in 2009**		**Extraction method**		**PCR assay**	**PCR target**
1	10/10 (100)		0 (0)		0 (0)		100–500		Commercial		Real-time	IS*2404*, IS*2606* and KR
4	5/10 (50)		1 (10)		4 (40)		<100		Commercial		Real-time	IS*2404* and KR
5	10/10 (100)		0 (0)		0 (0)		100–500		Commercial		Real-time	IS*2404* and ER
7	9/10 (90)		0 (0)		1 (10)		100–500		Commercial		Real-time	IS*2404* and KR
8	10/10 (100)		0 (0)		0 (0)		<100		In-house		Real-time	IS*2404*, IS*2606* and KR
9	10/10 (100)		0 (0)		0 (0)		<100		Commercial		Real-time	IS*2404*
10	8/10 (80)		2 (20)		0 (0)		<100		Commercial		Real-time	IS*2404*
11	7/10 (70)		0 (0)		3 (30)		<100		Commercial		Nested	IS*2404* and IS*2606*

a The total n° of suspensions analyzed varies per laboratory because some laboratories reported inconclusive results. ^b^ This laboratory did not formally submit results, but used the program to troubleshoot its DNA extraction and PCR protocols with the assistance of the coordinating laboratory (see results for details). ER: enoyl reductase domain; KR: ketoreductase B domain.

In the first round, one laboratory reported false positive results (Table 5). In the second round, one laboratory reported false positive results, two laboratories reported false negative results and one laboratory reported both false positive and false negative results.

#### Laboratory type, workload and methods used

In both rounds, the majority of participants were reference or academic laboratories. Three laboratories reported testing between 100 and 500 environmental samples per year while the other participants tested less than 100 samples. As with the clinical EQAP, participants used a range of DNA extraction protocols, PCR methods and amplification targets, including both in-house methods and commercial kits for DNA extraction and gel-based and real-time PCR for sequence detection. However, in both rounds, commercial kits were most commonly used for DNA extraction and real-time PCR targeting IS*2404* (+/− other targets) was most commonly used for sequence detection. There was no correlation between participants’ performance and laboratory type, workload or method. The two panels retained by the coordinating laboratory and tested after all participants had submitted results yielded results consistent with the expected results (in most cases less than a 10-fold difference in detection of *M. ulcerans* DNA), demonstrating that any delay in testing was unlikely to have been a cause of discordant results (Table 3).

## Discussion

The results of the two rounds of clinical and environmental EQAP indicated that there is a great variation between laboratories in the quality of molecular detection of *M. ulcerans* from clinical and environmental samples. Only 36% of the laboratories in the first round of the clinical EQAP and 31% in the second round had more than 90% concordant results. In both environmental rounds, however, at least half of respondents reported 100% concordant results. This discrepancy between the clinical and environmental EQAP could be explained by the fact that, in the environmental program, samples had a higher bacillary load and because the laboratories participating in the environmental EQAP were among the stronger ones in the clinical EQAP (87% and 90% concordant results in the first and second round respectively).

False positive results indicate problems of specificity and are suggestive of cross-contamination during DNA extraction or PCR most often due to carryover amplicon contaminations from previously amplified PCR products. This highlights the importance of the three-room PCR principle (where DNA extraction, mastermix preparation and template addition are separated) and reinforces the need for performing DNA extraction from clinical (particularly cultured isolates) and environmental samples in separate areas with dedicated reagents and equipment. The false negative results may be due to poor DNA extraction efficiency, low PCR sensitivity and/or PCR inhibition.

Among the nine laboratories with discordant results during the first clinical EQAP, four had both false positive and false negative results indicating both problems of sensitivity and specificity of their DNA extraction and/or PCR assay. The laboratories with few concordant results all had low intra-laboratory reproducibility indicating that their false results were probably due to mistakes in manipulations rather than to the techniques used. This impression was reinforced by the observation that every extraction method and PCR assay showed great variations in concordant results. Moreover, reference laboratories had more concordant results than academic and hospital laboratories, again suggesting that the variation was most probably due to laboratory performance.

In the second round of the clinical EQAP five laboratories had an intra-laboratory reproducibility of more than 90% with three of them processing more than 100 clinical specimens in 2010. Among the laboratories with false results, a majority of 13 had false negatives indicating problems with the sensitivity of DNA extraction and/or PCR assay. Six of these laboratories had intra-laboratory reproducibilities over 90% indicating that their false results were due to the techniques in use rather than to manipulation errors.

Indeed, the two laboratories with the lowest number of concordant results (lab05 and lab15) did have high intra-laboratory reproducibilities. Moreover, they reported no false positive results suggesting that the low sensitivity was probably due to a problematic PCR assay rather than manipulation errors. They both indeed did not detect less than 29×10^−5^ ng/µl DNA.

For most laboratories the limited sensitivity of molecular detection was probably due to a weak detection by the PCR assay as demonstrated in Fig 3. Five laboratories detected DNA dilutions up to 29×10^−6^ ng/µl only and also reported several false negative results among the set of specimen suspensions. Among the laboratories that detected 29.10^−7^ ng/µl DNA some still reported several false negative suspensions indicating the use of an insufficiently sensitive DNA extraction method. Manipulation errors may also explain the combination of false positive results and a low intra-laboratory reproducibility (lab02, lab23 and lab28).

More laboratories participated in the second round of the clinical EQA program (11 in 1st vs. 18 in 2nd round) and the median proportion of concordant results increased (from 82% to 88%). Also the median inter- and intra-laboratory reproducibilities increased respectively from 90% to 94% by sample and from 88% to 94% by laboratory. The number of laboratories reporting false positive results reduced from 64% to 38% while the number reporting false negative results increased from 55% to 81%, suggesting that the set of specimen suspensions distributed in the second round included some difficult ones/weak positives, challenging the detection limit of the PCR assays used. Eight laboratories participated in both rounds. Four laboratories performed better in the second round while three performed worse. One laboratory twice reported 100% concordant results. One of the laboratories that performed worse in round 2 (coded lab08 in round 1 and lab05 in round 2) reported results with a reduced concordance but a higher intra-laboratory reproducibility (Table 3). This laboratory reported many false negatives and did not detect more than 29×10^−5^ ng/µl *M. ulcerans* DNA indicating that solving the PCR sensitivity problem will probably increase the number of concordant results compared with the 1st round. In a repeated quality control study for the molecular detection of *M. tuberculosis*, improved performance was observed as well [15], [16].

Three of the eight laboratories that participated twice in the clinical EQA program used a different method for either DNA extraction (a commercial instead of an in-house method) or amplification (qPCR instead of a single run and nested conventional PCR assay). The laboratory that changed to a commercial DNA extraction method slightly increased its performance with less false positive but an increase in false negatives. Both laboratories that changed to real-time PCR as their amplification method had reduced performances with more false positives as well as false negatives. This could be due to those laboratories not being acquainted yet with the newly implemented technique.

Both in the clinical and environmental EQAP the laboratories’ performances did not correlate with the methodologies used for DNA extraction and amplification. It is therefore not possible, based on the results presented here, to make any recommendations on methodologies. Comparisons of different methods for the molecular detection of *M. ulcerans* in clinical and environmental specimens have been described previously [20], [22], [25].

Of the five laboratories with false results in either the first or second round of the environmental EQAP, two reported false positive results, two reported false negative results and one reported both false positive and false negative results. In the second round, the three positive samples that were incorrectly reported as negative by at least two laboratories were the types of samples that typically contain PCR inhibitors (faeces and soil). The fact that one of these samples had an IS*2404*-Ct value of 22.19 (the strongest positive sample in the panel) and that these laboratories correctly detected *M. ulcerans* DNA in the water sample that had a IS*2404*-Ct value of 36.19 (the weakest positive sample in the panel), suggests that these false negative results were due to inhibition (although none was reported by participants) rather than low sensitivity of their DNA extraction or PCR assay. These results highlight the importance of including internal positive controls in every reaction to test for inhibition and the challenge of optimizing DNA extraction protocols and PCR assays to reduce inhibition without compromising sensitivity.

Implications of the false positive and false negative results of respondent laboratories may be that (i) patients suffering from illnesses other than BU receive inappropriate treatment; (ii) patients with BU are erroneously considered as suffering from other illnesses; (iii) epidemiological data on BU are unreliable; (iv) conclusions drawn from clinical as well as environmental studies are doubtful; (v) researchers pursue or abandon lines of research on the basis of incorrect environmental results, which hampers efforts to elucidate the mode of transmission and environmental reservoir of *M. ulcerans;* and (vi) PCR as a single confirmatory test is insufficient for laboratories with weak EQA results (<90% concordant results).

We therefore recommend that: (i) these quality assessment programmes are continued on a regular basis; (ii) laboratories investigate the areas for improving their performance; (iii) laboratories implement rigorous internal quality control procedures (with e.g. the inclusion of (weak) positive and negative controls in every DNA extraction batch and PCR run as well as the inclusion of an internal positive control in every reaction); and (iv) the diagnosis of BU by microscopy is reinforced. Two-thirds of PCR positive specimens can be confirmed by direct smear examination [8], [9], [26], [27]. Direct smear examination is a cheap and fast diagnostic method that can be applied easily in BU endemic areas without the need for expensive and sophisticated equipment [28], [29]. Moreover, in most BU endemic countries systems are in place to control the quality of direct smear examination by national tuberculosis programs, and the periodic (e.g. quarterly) correlation of smear microscopy results with PCR results can serve as an additional internal control to detect problems with the molecular diagnosis of *M. ulcerans*.
